# Supplementary material for: Human IgG and IgA responses to COVID-19 mRNA vaccines
Source: PLoS One. 2021 Jun 16;16(6):e0249499. doi: 10.1371/journal.pone.0249499 (PMC8208542; doi:10.1371/journal.pone.0249499)
Supplement: S1 Table — (PDF) [file pone.0249499.s001.pdf]

Human IgG and IgA responses to COVID-19 mRNA vaccines

Adam V Wisnewski*, Julian Campillo Luna*, Carrie A Redlich
Department of Internal Medicine, Yale University School of Medicine, New Haven, CT 06520

	IgG ELISA OD				IgA ELISA OD			
	Subject #				Subject #			
Days Post Vaccine	1	2	3	4	1	2	3	4
0	0.025				0.479			
17	1.558				2.878			
28	1.379				1.224			
38	2.850				2.846			
81	2.254				0.619			
146	1.39				0.39			
0		0.014				0.228		
5		0.011				0.235		
8		0.062				0.731		
10		0.216				1.205		
21		0.930				1.333		
28		1.113				0.774		
35		2.980				3.49		
73		2.286				0.941		
138		1.436				0.832		
3			0.052				0.213	
7			0.026				0.188	
15			1.769				1.06	
21			1.812				0.774	
27			1.813				0.375	
35			3.490				1.843	
40			3.490				1.142	
48			3.343				0.823	
131			2.29				0.258	
2				0.308				0.252
8				0.584				0.499
14				2.603				2.198
22				2.547				1.274
26				3.141				2.026
34				3.093				1.645
41				3.083				1.102
47				3.073				1.107
130				2.130				0.447
